# Current Updates on Expanded Carrier Screening: New Insights in the Omics Era

**DOI:** 10.3390/medicina58030455

**Published:** 2022-03-21

**Authors:** Iolanda Veneruso, Chiara Di Resta, Rossella Tomaiuolo, Valeria D’Argenio

**Affiliations:** 1 Department of Molecular Medicine and Medical Biotechnologies, Federico II University, Via Sergio Pansini 5, 80131 Naples, Italy; venerusoi@ceinge.unina.it; 2 CEINGE-Biotecnologie Avanzate, Via G. Salvatore 486, 80145 Naples, Italy; 3 Università Vita-Salute San Raffaele, Via Olgettina 58, 20132 Milan, Italy; diresta.chiara@hsr.it; 4 Department of Human Sciences and Quality of Life Promotion, San Raffaele Open University, Via di Val Cannuta 247, 00166 Rome, Italy

**Keywords:** genetic carrier screening, expanded carrier screening, next generation sequencing, recessive genetic disease, genetic counselling

## Abstract

Genetic carrier screening has been successfully used over the last decades to identify individuals at risk of transmitting specific DNA variants to their newborns, thus having an affected child. Traditional testing has been offered based on familial and/or ethnic backgrounds. The development of high-throughput technologies, such as next-generations sequencing, able to allow the study of large genomic regions in a time and cost-affordable way, has moved carrier screening toward a more comprehensive and extensive approach, i.e., expanded carrier screening (ECS). ECS simultaneously analyses several disease-related genes and better estimates individuals’ carrier status. Indeed, it is not influenced by ethnicity and is not limited to a subset of mutations that may arise from poor information in some populations. Moreover, if couples carry out ECS before conceiving a baby, it allows them to obtain a complete estimation of their genetic risk and the possibility to make an informed decision regarding their reproductive life. Despite these advantages, some weakness still exists regarding, for example, the number of genes and the kind of diseases to be analyzed and the interpretation and communication of the obtained results. Once these points are fixed, it is expectable that ECS will become an ever more frequent practice in clinical settings.

## 1. Introduction

Genetic carrier screening (GCS) means to identify DNA variants associated with specific genetic diseases and to provide information regarding the risk of transmitting them to the offspring, exploiting molecular tests in apparently healthy individuals. Consequently, GCS and relative counseling should be ideally suggested and provided to couples before pregnancy to obtain timely information regarding their reproductive risk and explain them the available reproductive options [1,2]. GCS was used during pregnancy for the first time in 1970 to identify DNA variants related to hemoglobinopathies onset [3]. Subsequently, it has been extended to an increasing number of inherited conditions (i.e., spinal muscular atrophy, cystic fibrosis, Tay Sachs disease, fragile X syndrome, and many others) so that a few years ago, the Committee on Genetics of the American College of Obstetricians and Gynecologists (ACOG) published general GCS recommendation and specific guidelines for some most common genetic diseases [1]. Moreover, specific considerations were also provided for ethnic groups having a higher risk of such conditions. Indeed, the probability of being a carrier of a genetic condition is influenced by both ethnicity and family history [3]. Based on this evidence, GCS has been traditionally proposed to individuals/couples just for a specific disease based on the presence of an estimated risk depending on a positive family history and/or on a high risk in certain ethnic groups.

This approach has been recently overcome by a more enlarged one. Indeed, the availability of molecular techniques able to analyze genomic regions with affordable costs has allowed the diffusion of the so-called Expanded Carrier Screening (ECS). ECS means using molecular techniques to simultaneously analyze multiple DNA mutations or genes responsible for several genetic diseases without a clear risk of inheritance in the individual/couple admitted to the test. In this way, it is possible to avoid biases related to incomplete familial information or difficulties related to the correct estimation of the ethnic risks, also considering the multiethnicity of modern societies. However, more data mean more information, including the so-called incidental findings and the consequent need to finely regulate ECS and the following communication of the obtained results [4].

This review aims to summarize the current state-of-the-art regarding ECS use. In particular, not only ECS’s benefits and risks will be analyzed, but we will also focus on methodological aspects and ethical issues related to the use of this kind of molecular test.

## 2. Materials and Methods

PubMed search was carried out to search for indexed articles in English regarding ECS. The following keywords were used: “genetic carrier screening”, “expanded carrier screening”, “carrier identification”, “preconception carrier screening”, and “NGS and carrier identification”. The temporal window for the research was fixed between 2001 to date to focus on the most recent publication in the field; a manual search for older but significant references cited in the reviewed articles was made when appropriate.

## 3. Expanded Carrier Screening: Definition, Pros and Cons

As mentioned above, ECS is the simultaneous analysis of a variable in size panel of DNA mutations/genes to assess the carrier status for multiple genetic diseases at a time. Already in 2015, Nazareth and colleagues pointed on this changing trend in molecular genetics due to technical implementations on one side and the reduction of molecular tests’ costs on the other [5]. Accordingly, a couple of years later, the ACOG committee published an opinion on the use of carrier screening in the genomic era, including ECS within the acceptable strategies [6]. Since the costs of molecular tests are constantly decreasing, it is conceivable to suppose that ECS will become even more frequently offered. Moreover, considering that rare genetic diseases occur in 1 in 280 births [7,8], the routine use of ECS, allowing the simultaneous risk assessment for a wide spectrum of genetic diseases, such as hemoglobinopathies, cystic fibrosis, Tay Sachs disease, fragile X syndrome, spinal muscular atrophy, Canavan disease, familial dysautonomia, and many others [3], could have significant benefits. First, concerning traditional approaches, ECS is not based on ethnicity-based risks; thus, it overcomes the possibility of missing a diagnosis based on epidemiological data on disease frequency in a certain population [3,5]. In addition, the use of ECS irrespective of ethnicity will allow a better estimation of carriers’ frequency and, consequently, of diseases risks also in couples whose partners have different ethnic backgrounds. In turn, the analysis of a high number of diseases in a high number of individuals will improve couples’ management, allowing a better evaluation of their reproductive options [9]. Of course, to maximize these advantages, ECS should be carried out on both partners for having a more accurate estimation of the couple’s risk to transmit an inherited disease to their newborns [10]. However, the debate on this point is still open. Janssens et al., reporting the opinion of a panel of European geneticists on ECS, highlighted that even if offering this test to couples increases its clinical utility, especially in the case of couples requiring assisted reproduction procedures, the individual screening allows to enlarge the analysis to other family members, identifying the at-risk individuals [11]. Punji et al. designed a preconception, sequential carrier screening based on genome sequencing to analyze 728 disease-related genes in 131 women planning a pregnancy [12]. They found that 102/131 analyzed women were carriers of at least one genetic mutation; interestingly, only 71/102 male partners accepted to carry out the test and were successively screened [12]. A patients-based survey to understand the reasons for declining ECS highlighted that the most common causes were lack of time or interest, underlying a variable interest in performing this test and the need to take into account the individual choices [13]. Similarly, Plantinga et al. carried out an online survey in the Netherlands to verify people’s opinion about a preconception ECS for couples developed to evaluate 50 inherited diseases simultaneously: within the 504 responders, only 34% would like to undertake ECS [14]. Taken together, these studies highlight that, besides the scientific societies’ recommendations regarding the ECS offering to couples rather than individuals, individuals’ needs and choices must be taken into account; above all, it is crucial to respect the union of the couple in reproductive choices [15]. In this context, education programs and detailed counselling may provide further instruments to people supporting them in making their informed decisions.

In addition to the above, timing is another crucial factor. Ideally, ECS should be performed before pregnancy to avoid pressures related to the progress of the pregnancy itself. Indeed, there is a consensus among scientific societies that the ideal time for ECS is preconception. Both partners can perform it simultaneously or sequentially even if concomitant testing should be preferred to limit time constraints [9]. In this context, pre- and post-test counseling should always be provided to furnish explanations regarding the kind of information that the test may provide, its limits, and the options available based on the estimated genetic risk [3,5]. Nevertheless, ECS is still not routinely offered to couples planning a pregnancy.

A retrospective study involving more than 340,000 individuals showed that, in populations with mixed races, ECS might be useful to increase the detection rate of carriers for several genetic diseases [16]. A subsequent study was carried out by retrospectively analyzing 123,136 exomes from different ancestries to estimate the carrier frequencies for 415 genes associated with recessive diseases, confirming the efficacy of ECS in increasing the diagnostic yield of carrier status detection in prospective parents [17]. However, by stratifying the results based on ancestry, they pointed out a matter still up for debate: which is the number of conditions and/or genes to be included in the ECS? Based on their data, Guo and colleagues estimated that using as threshold a carriers’ rates > 1%, an ancestry-specific panel including 5 up to 28 genes (based on the ethnicity) or a pan-ethnic panel including 40 genes give results similar to the exome sequencing in terms of carriers’ detection [17]. On this aspect, indeed, no consensus still exists on the diseases that ECS should include [9]. The ACOG committee just highlighted the need of using well-designed genetic panels able to increase the carriers’ detection rate and, at the same time, avoid the analysis of un-useful genomic loci and recommended that each disease included in an ECS panel should have some of the following criteria: a clear phenotype, a negative impact on patients’ quality of life, development of mental or physical retardation, needs for surgical or medical interventions, early-onset, availability of prenatal diagnosis, and/or a carrier rate equal or greater than 1-in-100 [6]. Thus, considering the high number of rare diseases that could be potentially screened, their different population-based frequencies, and the absence of specific criteria for genes selection, currently, ECS panels greatly vary across different laboratories, each offering its own panel. In the attempt to compare the different available ECS panels, in 2017, Chokoshvili and colleagues identified 16 ECS providers. Interestingly, they highlighted remarkable differences in the number of screened diseases, ranging from 41 to 1792, with just 3 (cystic fibrosis, maple syrup urine disease 1b, and Niemann–Pick disease) being included in all the 16 panels, underlining the importance to establish clear criteria for ECS panel design [18]. Similarly, Stevens at al. highlighted that several conditions included in the currently used panels did not match the ACOG proposed criteria; thus, even if ECS benefits are recognized, they underline once again the need for collaborative efforts to design clinically useful panels reducing patients’ stress and unnecessary tests’ costs [19]. In this context, Ben-Shachar et al. carried out a data-driven evaluation to estimate the size and content of ECS gene panels [20]. By retrospectively analyzing 56,281 patients, they verified if, for each of the 176 tested diseases, the above-mentioned ACOG criteria [6] were respected. Their results show that the threshold of the 1-in-100 carrier rate is not adequate in the correct identification of the at-risk couples and suggested that the clinical detection rate of a disease may be an alternative measure [20]. Based on all the above, in an intriguing commentary with the iconic title “Expanded carrier screening: how much is too much?”, Wapner and Biggio pointed out some dual aspects of ECS [21]. Indeed, if it is true that ECS represents the possibility of identifying the carriers of most Mendelian diseases and, hopefully, preventing diseases’ onset by applying subsequent preconception and/or prenatal diagnostic procedures, it also represents a time- and money-consuming procedure associated with a high level of responsibility. Thus, the choice of the conditions to analyze by ECS should be based on these considerations and should include the diseases matching the ACOG criteria and for which prenatal diagnosis is also available to optimize couples’ reproductive outcomes and perinatal health. Despite this, the current trend is to continuously increase the number of tested rare diseases since the selling companies use the number of genes included in the ECS panel as an advantageous feature over competitors. This phenomenon increases the difficulties in interpreting test results and the consequent patients’ management, enhancing the need for genetic counseling and increasing couples stress and anxiety [21]. Considering all the above, the authors provocatively state that, in the era of personalized medicine, maybe patients should be the stakeholders, and ECS panel content should be defined based on patients’ expectations and needs [21]. In the attempt to define some of these points and answer to commonly asked questions, the American College of Medical Genetics and Genomics (ACMG) recently published a practical resource for ECS proposing a tiered approach to be assessed in the context of family and personal history to evaluate the analysis of additional genes [22].

Another issue is related to marketing aspects since ECS is often sponsored directly to citizens even if, in most cases, to access the test, a request by a physician is required [9]. As already mentioned, several studies carried out to evaluate the general population interest in ECS have highlighted that the lack of interest is due to both awareness and knowledge [23]. It has to be underlined that, with respect to selected at-risk groups, the general population is less familial with genetic diseases and tests, affecting the ECS acceptance rate. Moreover, it has also emerged that the physicians themselves are often not familiar with ECS, highlighting the need for professional education regarding who to admit to the test and when and how to interpret the results.

**Figure 1:**
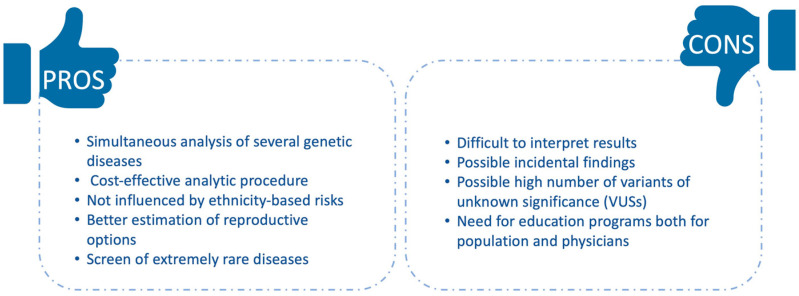
Pros and cons of ECS testing.

Even if these aspects need to be assessed to regulate ECS application, it has shown many advantages (Figure 1).

It is undeniable that ECS is becoming an even more frequent procedure, and technical advancement has made it more cost effective than traditional “targeted” options [9]. However, its implications have to be carefully evaluated to maximize ECS benefits. The definition of clear guidelines is desirable and may drive, in the future, more appropriate use of ECS, supporting its future implementation in routine clinical practice.

### 3.1. Technical Issues

ECS has been prompted by recent technological advancements that have made it possible to analyze several genomic loci/genes in a time- and cost-affordable manner [4]. Moreover, the current ECS development and diffusion has been driven by the observation that, although ECS screened diseases are individually rare, the cumulative risk of having an affected baby is not so rare, especially if compared to other conditions commonly screened during pregnancy [5]. Nevertheless, ECS offering is extremely variable in terms of panels size and used technologies, making it difficult to compare results from different laboratories in addition to confusing patients [24].

The first method used to design ECS panels was targeted genotyping [5]. Targeted genotyping uses array-based technologies to specifically analyze hundreds or thousands already known DNA variants through specifically designed probes [25]. Almost ten years ago, Lazarin et al. reported the use of a mutations panel able to simultaneously analyze more than 400 Mendelian variants associated with more than 100 diseases: by analyzing 23,453 individuals with different ethnicities, they found that 24% carried at least one mutation, supporting the routine use of pan-ethnic ECS [26]. However, targeted genotyping is limited to a set of known disease-causing variants for each tested condition; thus, this approach may lack diagnostic sensitivity and accuracy since rare or novel mutations will be undetectable. Moreover, mutations frequency varies across different ethnic groups so that carrier detection may be impaired based on patients’ ancestry. This aspect in turn may make it difficult to estimate the individual residual risk in case of a negative result [5].

The recent development of next-generation sequencing (NGS)-based approaches and their validation in routine diagnostic settings [27,28,29] has enhanced the use of NGS-based panels for ECS purposes. Indeed, NGS technologies have been assessed as sensitive and accurate methods for nucleic acids sequence analysis able to detect different kinds of DNA alterations (both known and unknown) and to simultaneously analyze large genomic regions at reduced cost in respect to other techniques [30]. Bell et al. reported the evaluation of a preconception, NGS-based ECS to test for 448 severe, recessive diseases in 104 unrelated subjects: they found an average carrier burden of 2.8 and, based on this finding and the test’s lower cost compared to lifetime diseases’ treatments, suggested that NGS-based ECS should be offered to the general population to impact the incidence of rare recessive disorders [31]. Hallman et al. carried out a clinical validation of a NGS-based ECS and, by analyzing 11,691 preconception patients, identified 447 carriers and two affected subjects; interestingly, about 25% of the identified mutations were rare mutations not included in traditional tests, thus underlying the advantage of NGS over traditional approaches [32]. A study carried out on 805 individuals allowed to identify 352 mutations carriers (43.7%), thus suggesting that all couples who wish to conceive should consider NGS-based ECS irrespective of their ethnicity [33]. In this regard, Westemeyer et al. reported the use of NGS-based ECS in the general U.S. population; by analyzing 381,014 individuals, they were able to provide more information compared to traditional screening, thus suggesting the use of NGS to carry out the ECS in all reproductive-age women [34]. 

NGS-based panels for ECS also allow estimating carriers’ frequency in less-studied populations/ethnic groups. Singh et al. used a targeted NGS based approach to analyze 200 unrelated individuals from northern India; they found a carrier frequency of 26% and also highlighted that most of the pathogenic variants identified were different from those commonly found in the West, thus underlying the advantage to use NGS [35]. Similarly, Chan et al., using a NGS-based ECS to evaluate 123 infertile Chinese women, found that 58.7% of the tested individuals were carriers of at least a disease and suggested the use of ECS to better estimate carrier frequency in Chinese people [36].

Finally, to overcome the already discussed controversy related to different gene panels size, Punj et al. evaluated the possibility to use genome sequencing for ECS purposes: they found that this approach has higher diagnostic sensitivity with respect to the NGS-based targeted ones even if variants interpretation may be challenging [12].

The main features of all the studies reviewed herein are summarized in Table 1 to allow their easier comparison.

Overall, the use of NGS has the advantage to allow both pan-ethnic screening and the simultaneous analysis of an increasing number of diseases [9]. Indeed, with respect to the mutation-based genotyping panel, often specific for some ethnic groups, NGS can also identify rare and/or novel mutations. However, it has to be mentioned that most of the commonly offered ECS panels do not include intronic regions [25]. Moreover, some other limitations of these technologies have to be taken into account, such as the possible lack of mosaicisms due to low sequencing coverage, the need for high data storage, and the identification of a large number of variants of unknown significance (VUSs) [9,12]. Currently used NGS methodologies are not able to detect all the possible DNA variants, such as triplet repeats, and short reads may be challenging for highly homologous genomic loci [9,12]. Finally, many inherited diseases are featured by high clinical variability due to variable expression and penetrance, so disease prediction later in life based just on genotype may be very tricky [9].

Within the NGS-based approaches, genome sequencing has the advantage to avoid biases that may occur during libraries preparation procedures, such as PCR biases or a not homogeneous representation of the target regions, and may allow a better estimation of structural variants [12]; however, the above-mentioned pitfalls related to the high quantity of data generated and their difficult interpretation will be still more represented. It is to be expected that, as has already happened with NGS about ten years ago, the advent of the so-called third-generation sequencers, thanks to long reads implementation, will provide a solution to some of these biases [37]. Moreover, novel bioinformatic tools will allow structural variations detection based on sequencing data increasing ECS sensitivity [38]. In this context, a very recent publication by Zhao et al. reported the validation of NGS for the detection of *SMN1* gene copy numbers by analyzing 478 samples with multiplex ligation probe amplification (MLPA), real-time quantitative polymerase chain reaction (qPCR), and NGS; interestingly, they found that NGS performed better than the other two methods and suggested that it can be useful in ECS context to reduce the need for multiple methodologies and, in turn, analysis time and costs [39].

These technical innovations will open the way to novel methodologies to improve currently available ECS.

### 3.2. Ethical Issues

As mentioned above, ECS allows to simultaneously analyze an increasing number of diseases in individuals and/or couples to identify the “at-risk” subjects, provide them information regarding their risk of transmitting a disease to the offspring, and discuss their consequent reproductive options. As is easy to expect, using this test in a clinical context raises several ethical concerns [40].

First of all, pre- and post-test genetic counseling should always be provided, but this is difficult to set up in the case of population-based screening. Accordingly, scientific societies have stated that, in this context, the pre-test counseling is not only not practical, but also unnecessary [41], and it has also been proposed that traditional genetic counselling could be reserved just for difficult cases, while in a routine setting, pre-test counseling could be entrusted to other medical professionals [5]. This implies a continued medical education to ensure updated knowledge on a rapidly evolving field. 

Education programs should also be provided to the general population to minimize anxiety, avoid stigmatization related to genetics, and increase the rate of ECS acceptance. However, it has been already reported many years ago that anxiety related to carrier status seems to be overestimated since it disappears in a few months unless a positive family history and an affected child is already present [42,43]. Accordingly, based on a survey carried out on 240 women planning a pregnancy who refused ECS, Gilmore et al. found that most of them declined the enrollment for lack of interest/time, while a minority stated they do not want to have this information or are afraid of getting stressed [13]. However, further, well-designed studies are required to define the long-term effects of ECS on individuals’ well-being.

While pre-test counseling might be avoided, post-test counseling is crucial to discuss ECS results. Indeed, if the test has been carried out as a preconception screening or during pregnancy, the obtained information could support couples in making informed decisions regarding their reproductive options and gynecologists in properly monitoring pregnancy and delivery. In this context, it has to be mentioned that some of the diseases included in the ECS panel are extremely rare, and their causative mutations, related phenotypes, and long-term outcomes might be largely unknown. This impairs counselling efficacy both in the case of positive results since it does not allow a correct risk estimation and in the case of a negative one since it makes it difficult to evaluate the residual risk.

A particularly critical point is represented by the difficulty to a priori assess the clinical severity of a disease. Considering the enlarged and inclusive design of the currently offered ECSs, really uncommon diseases, whose severity may be not clearly recognized, may be identified, impairing pre-test and, mostly, post-test counseling [5]. This situation could be far from rare given that, while diseases prevalence is low, mutations carriers’ rate is high in different populations. To support health care professionals in this task, a disease-severity classification in four tiers has been proposed [44]. Even if this kind of classification may somewhat facilitate communication, it does not consider the individual variability and the variable phenotypic expression and penetrance that the same disease, sometimes in the same family, can present [9]. Moreover, some disorders may have a mild phenotype that does not justify any intervention but raises questions about their inclusion in the ECS panel. Similar considerations may apply to late-onset diseases, especially if treatments are available.

Another weakness is represented by the potentially high number of VUSs identified in each analyzed subject. Patients should be informed about the possibility of obtaining uncertain results and that variants’ significance interpretation and classification may vary over time [3].

Finally, in the case of negative results, it is crucial to underline that this does not mean having any risk. Still, several factors can influence residual risk, including technical factors, current knowledge, or individual factors [3]. 

All the above-mentioned issues have to be carefully taken into account, especially if we consider the potential consequences that the indiscriminate diffusion of enlarged genetic tests, such as ECS, may have at political level and within specific communities. In this context, the Dor Yeshorim project has to be mentioned. It is a premarital carrier testing program implemented in an ultra-orthodox Jewish community with the aim to reduce the number of children affected by genetic diseases; as expected, this program is raising an intense debate on the limitations regarding the personal freedom and on its coercive aspects [45]. A similar program is compulsory also for couples before they get married in the United Arab Emirates limited to hemoglobinopathy carrier status assessment and justified by the high prevalence of this disease in that population [46]. These experiences, currently limited to a single disease or to restricted ethnic groups, highlight once again the need for clear guidelines to regulate the use of such genomic testing procedures.

## 4. Conclusions

**Figure 2:**
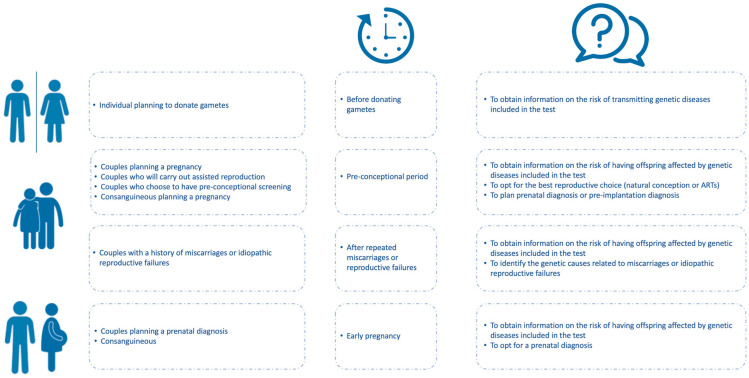
Indications for Expanded Carrier Screening (ECS) planning. ECS has potential benefits in different conditions if properly used. Who is admitted to the test, when it should be preferably carried out, and which kind of information it can provide are summarized herein, underlying its potential advantages in routine diagnostic settings.

ECS has the potential to allow an extensive evaluation of individuals’ carrier status independently from their genetic, familial, or ethnic backgrounds. This has the advantage of making individuals aware of their risk of transmitting a genetic disease and making timely, informed decisions regarding their reproductive life (Figure 2). 

Moreover, currently available technologies make ECS a time- and cost-efficient molecular test. However, besides different ECS panels being sold on the market, their use in the clinical context is still incomplete. As discussed in the previous sections, several points need to be addressed to improve people’s and healthcare professionals’ perception and acceptance of ECS. Once these pitfalls are fixed, ECS benefits will support an ever more capillary diffusion of this kind of test.

## Figures and Tables

**Table 1:** Comparative table reporting the study design and the main findings of the cited ECS studies.

Study	ECS Methodology	DNA Variants/Genes Panel	Studied Population	Main Findings
Lazarin et al. [26]	Targeted genotyping	417 DNA variants associated with 108 diseases	23,453 individuals with different ethnicities	24% of individuals carried at least one condition
Bell et al. [31]	Targeted NGS	437 genes	104 unrelated subjects	Average carrier burden of 2.8
Hallam et al. [32]	Targeted NGS	15 genes	11,691 preconception patients	447 carriers and 2 affected subjects individuated; 25% were rare mutations not included in common preconception tests
Hernandez-Nieto et al. [33]	Targeted NGS	283 genes	805 individuals (391 of which are couples)	352 carriers (43.7%)
Westemeyer et al. [34]	Targeted NGS	274 genes	381,014 individuals with different ethnicity and age	1 in 44 (2.3%) couples was at risk for genetic disorder
Singh et al. [35]	Targeted NGS	88 genes	200 unrelated individuals	52 carriers (26%)
Chan et al. [36]	Targeted NGS	104 genes	123 infertile women and 20 of their partners	58.7% were carriers
Punj et al. [12]	Genome sequencing	728 genes	202 individuals (131 women and 71 of their partners)	78% were positive carrier for at least one condition; 304 variants found
